# Optimization of UAE-NADES green extraction of bioactive compounds from chickpea (*Cicer arietinum* L.) sprouts using simplex lattice mixture design methodology

**DOI:** 10.1016/j.ultsonch.2024.107186

**Published:** 2024-11-30

**Authors:** Waseem Khalid, Hyrije Koraqi, Imed E Benmebarek, Andrés Moreno, Tawfiq Alsulami, Robert Mugabi, Gulzar Ahmad Nayik

**Affiliations:** a Department of Organic Chemistry, Faculty of Chemical Sciences and Technologies, University of Castilla La Mancha, 13071 Ciudad Real, Spain; b Faculty of Food Science and Biotechnology, UBT-Higher Education Institution, Pristina-Kosovo; c Department of Food Science & Nutrition, College of Food and Agricultural Sciences, King Saud University, Riyadh 11451, Saudi Arabia; d Department of Food Technology and Nutrition, Makerere University, Kampala, Uganda; e Marwadi University Research Centre, Department of Microbiology, Marwadi University, Rajkot, Gujarat 360003, India; f Department of Molecular Food Chemistry and Food Development, Institute of Food and One Health, Gottfried Wilhelm Leibniz University Hannover, Hannover, Germany

**Keywords:** Chickpea sprouts, Novel extraction, Mixture design-simplex lattice, Antioxidants polyphenol content

## Abstract

In the present study, a statistical tool called the simplex lattice mixture design method was used to create a new formulation of Natural Deep Eutectic Solvent (NADES), which is derived from a combination of three compounds (citric acid, glycerol, and water) to extract bioactive compounds from chickpea (*Cicer arietinum* L.) sprouts. The mixture (natural deep eutectic solvent) was formulated by combining three solvents including citric acid, glycerol, and water. The extraction was performed in a sonication bath for 30 min. The simultaneous optimization was performed to obtain the highest total polyphenol content (TPC), total flavonoid content (TFC) and antioxidants activity. The highest values of total polyphenol content (TPC), total flavonoid content (TFC) and antioxidant activity were 128.0 ± 0.2  mg GAE/100 g, 38.61 ± 0.03 mg CE/100 g and 2117 ± 1.8 µmol TE/100 g respectively. HPLC-DAD of the optimized extract was utilized for quantification of polyphenol compounds showing catechin as the main compound followed by chlorogenic acid, epicatechin, syringic acid, rutin, gallic acid, kaempferol 3-glucoside, ferulic acid, and coumaric acid. These findings may represent a significant advancement in the management of phenolic compound extraction for targeted uses, such as serving as alternatives to traditional antioxidants primarily employed in the food industry to improve nutritional quality. Furthermore, our research has shown that mixture designs are an efficient and useful method for structuring and optimizing experimental parameters to achieve the most accurate results with the minimum number of experiments.

## 1 Introduction

*Cicer arietinum L.* is commonly known as a chickpea that belongs to the legume family and is composed of indispensable culinary and nutritional significance in the world [1]. There are different countries (Canada, India, Myanmar, Pakistan, Turkey, Ethiopia, Iran, México, Australia and the USA) that play major role in the production of chickpea [2]. However, it is mostly cultivated in the Northwest of Mexico. The chickpea can be sorted on basis of size, shape and color [3]. Moreover, the chickpea is good source of phytochemicals including amino acids, fatty acids, polysaccharide, phenolic compounds and antioxidants [4], [5].

Sprouts are nanoparticles that are visible when seeds start to grow. Sprouting is an economical, efficient and productive approach to seed processing that augments the nutritional and nutraceutical quality [6]. There are different types of sprouts including legume sprouts, cereal sprouts, grain sprouts and vegetable sprouts. However, there are various types of legumes present in nature. Moreover, different kinds of legume sprouts can be grown [7]. Various studies showed that sprouts contained more phytochemicals as compared to their grains. These phytochemicals play a vital role as anti-inflammatory, anticarcinogenic, antioxidant, antidiabetic, hypolipidemic agents in biological systems [8], [9]. Due to high concentration of phenolic compounds and their solvent interactions make it perplexing to identify the optimum extract technique [10], [11].

Phenolic compounds from plants are considered a vital component of the human diet and exhibit extraordinary antioxidant activity as well as other health benefits. From a structural perspective, the characteristic feature of a polyphenolic compound is an aromatic ring bonded to one or more hydroxyl groups. Phenolic compounds are generally considered natural antioxidants, critical in neutralizing free radicals and reducing the risk of various diseases. The antioxidant capacity of phenolic compounds depends on their molecular structure, such as the number and position of hydroxyl groups on the aromatic ring and the location of double bonds within the carbonic chain [12]. The number and position of the hydroxyl group in a specific phenolic compound led to a change in their antioxidant potential. These structural variations impact their antioxidant capabilities by affecting antioxidant pathways such as sequential proton loss electron transfer, single electron and proton transfer, and hydrogen atom transfer [13]. As a subclass of plant phenolics, phenolic acids have a resonance-stabilized structural and phenolic part, which donates a H atom and produces antioxidant properties through a radical scavenging mechanism. The antioxidant activity of phenolic acids is also known to occur through other mechanisms, such as singlet oxygen quenching and radical quenching via electron donation. Additionally, phenolic acids are widely distributed and have a long history of health benefits, including antibacterial, anticancer, anti-inflammatory, and anti-mutagenic properties [14].

Nowadays, researchers prefer to choose novel extraction methods as compared to conventional methods. However, ultrasound-assisted extraction (UAE) is one of the novel extraction techniques that is more systematized, productive and efficient as compared to conventional extraction techniques [15]. Ultrasounds facilitate the pre-treatment phase of extraction process, and significantly reinforce the extraction process. In addition, ultrasound-assisted extraction curtails the duration of extraction while escalating extraction productivity and efficiency by improving cavitation events like increased mass transfer rate, better solvent penetration in the cell and intensified mechanical mixing effects [16].

Furthermore, Natural Deep Eutectic Solvent (NADES) ensured the stability of extracted various bioactive compounds that may be potential applications in the formation of cosmetics, food manufacturing, and the production of pharmaceuticals [17], [18]. The drawbacks of conventional organic solvents include their high recovery energy consumption, toxicity to the environment, and volatility. Many researchers concentrate on creating green solvents as a solution to this issue [19]. However, NADES is an emerging green solvent that is entirely composed of natural components including sugars, organic acids, organic bases, etc. However, they are considered much safer and eco-friendlier than organic solvents [20]. For the preparation of NADES, hydrogen bond donor (HBD) and hydrogen bond acceptor (HBA) are generally incorporated to make strong hydrogen bonds, and create a low-temperature transition mixture [21]. Besides, NADES have desirable properties that are similar to ionic liquids including low vapor and high solubility [20]. Thus, it is more suitable for extraction process in food, pharmaceutical and cosmetic applications.

The simplex lattice design is designed for mixtures where the total percentages of the components reach up to 100 %. It is usually applied to areas that include a triangular domain [22]. The evaluation of the composition of the extraction mixture is crucial for the efficiency of the procedure. In one-sided studies, solvents with higher toxicity such as hexane and petroleum ether and exhaustive shaking were used [23]. The mixture design offers the following advantages: it requires a smaller number of experiments; it allows the study of the interaction between variables; and it is much more efficient in finding the best conditions for conducting the experiments [24], [25]. On the other hand, the mixture design, such as the simplex-lattice, allows the investigation of synergistic or antagonistic effects of the mixture components on the response variables [26].

An enhanced Simplex Lattice Mixture Design approach to investigate the impacts of combinations of the three natural chemicals called NADES for extracting bioactive compounds from the chickpea (*Cicer arietinum* L.) sprouts that are the focus of this study has not been reported in the literature. In order to predict an ideal combination characterized by its capacity to yield extracts from the sprouts under study that have the highest antioxidant activity and the most significant amounts of TPC and TFC, our work attempts to provide a useful method for designing and studying an effective, reproducible, and optimal model with the chosen properties. In this case, studies of the interaction between the sample matrix and solvent can be very helpful in the creation of ecologically friendly NADES-based methods. Given the above, the purpose of this work was to find the ideal solvent molar ratio for chickpea (*Cicer arietinum* L.) sprouts phenolic compounds, and antioxidant activity by using an experimental multivariate design to create an extraction method based on ultrasound-assisted extraction and Natural Deep eutectic Solvent (UAE-NADES). The efficacy of extraction and composition of the phenolic compounds extracted with NADES were determined by comparing them with a reference organic solvent. The other aim of this research was to enhance an extraction method for chickpea (*Cicer arietinum* L.) sprouts that was based on ultrasonic-assisted extraction with NADES for phenolic compounds, and antioxidant activity. The result of this study has implications for the developing environmentally friendly processes that could be applied in a number of industries, such as the food and pharmaceutical sectors. The extraction efficiency and solvent composition problems will also be resolved by this technique.

## 2 Materials and methods

### 2.1 Sample preparation

Chickpea grains were purchased from Mercadona, Cuidad Real, Spain. After that, chickpeas were washed with running water, and put in to tap water for 8 to 10 h. Furthermore, chickpea (*Cicer arietinum* L.) sprouts were placed in a sterilized plastic cartridge at 25 °C for the purpose of further processing. Then, wash chickpea sprouts after 24 h until 8 days. After 8 days, chickpea sprouts were harvested and put into freeze dryer at −80 °C for 48 h. However, freeze dried chickpea sprouts were grounded into fine powder (40 mesh) using Lab mill and stored at + 4 °C for further analysis.

### 2.2 Chemicals

All high-purity (> 99.0 %) substances (such as Trolox, gallic acid, glycerol, glucose, citric acid, maltose, lactic acid, Na_2_CO_3,_ HPLC-grade methanol, acetonitrile and formic acid) were utilized. All chemicals were bought from Sigma-Aldrich.

### 2.3 Preparation of NADES

NADES was prepared using the respected method of Koraqi et al. [27]. The NADES was formulated with different solvents including citric acid, water, and glycerol. Briefly, different solvents were mixed according to a specific molar ratio to synthesize NADESs. Then the magnetic stirrer was used to create the transparent, stable, and homogenous mixture at 70 °C. After that NADES was kept for cooling naturally at room temperature and stored in a dark place.

### 2.4 Experimental design

**Fig. 1:**
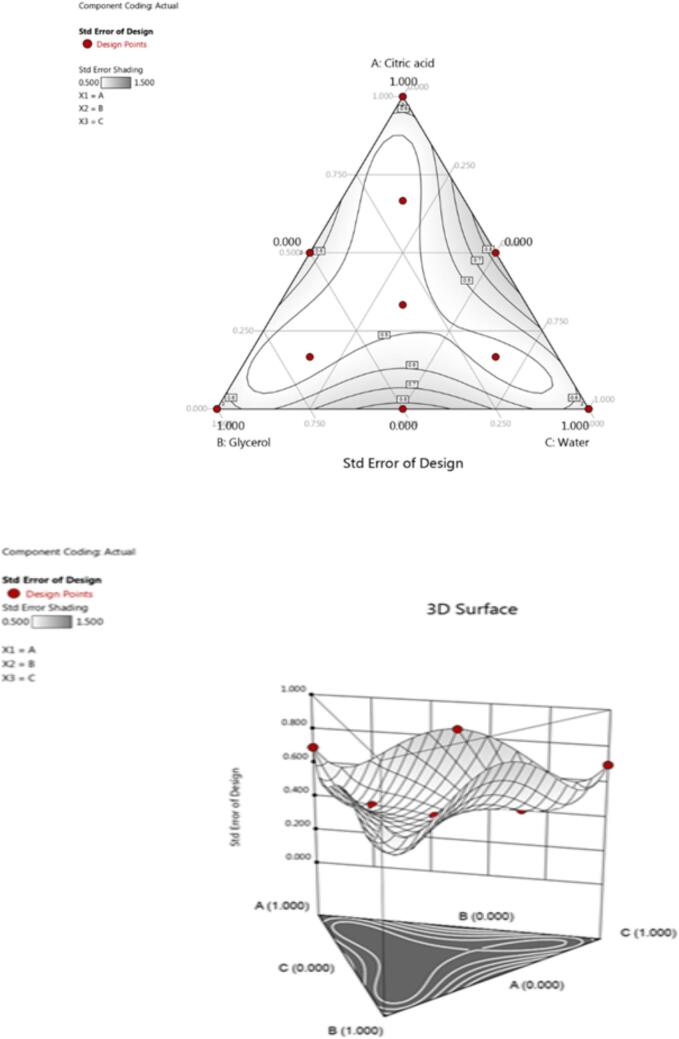
Architecture of the improved simplex lattice of the mixing solvents.

In order to determine the optimum solvent composition for extraction, simplex lattice mixture design was used. Based on available literature, three solvents were selected (water, glycerol and citric acid) in mixture design as independent variables. To find the best extraction solvent (solvent mixture), different responses were selected. Summary of used design is exhibited in Table 1. Component proportion was demonstrated as fraction of the mixture with a sum of one. Opted processing component along with their levels in each test run, along with 14 combinations of coded and un-coded experimental design are tabulated in Table 1 and Fig. 1. Simplex lattice mixture design analysis and plotting of the ternary plot were conducted with Design Expert® 13.0.

**Table 1:** Factor of solvent system mixed components.

Component	Name	Units	Type	Minimum	Maximum	Coded Low	Coded High	Mean	Std. Dev.
A	Citric acid (A)	%	Mixture	0	1	+0 ↔ 0	+1 ↔ 1	0.3452	0.3608
B	Glycerol (B)	%	Mixture	0	1	+0 ↔ 0	+1 ↔ 1	0.3452	0.3608
C	Water(C)	%	Mixture	0	1	+0 ↔ 0	+1 ↔ 1	0.3095	0.3690
			Mixture	Total = 0	Total = 1

### 2.5 Ultrasound treatments of chickpea (Cicer arietinum L.) sprouts

Ultrasonic treatment of chickpea sprout was developed based on the Muzykiewicz-Szymanska et al. [28] method with some modifications. Briefly, a quantity of 50 mg of fine powder of chickpea sprouts was introduced into an Erlenmeyer flask, followed by the addition of the prescribed volume of NADES solvent, which was 1 mL. The experiment was conducted using an ultrasound-assisted extraction (40 kHz). The mixture was sonicated for 30 min, then centrifuged to separate the supernatant at 3000 rpm for 10 min, and then filtered. The extract produced was stored at a temperature of 4 ◦C throughout all analyzes. The solvent used for the extraction of NADES was formulated from three components: citric acid, glycerol, and water according to the mixture design that was selected, the solvent fractions for each experiment were generated. All treatments were done in triplicate.

### 2.6 Total phenolic content (TPC)

The TPC of chickpea sprouts extract was measured using Folin–Ciocalteu method according to the procedure of Liu et al. [29] with minor modifications. Folin–Ciocalteu reagents were taken and diluted with distilled water. The sodium carbonate solution (about 70 g/L) was made using distilled water at pH (10). Then, 30 μL of sprout extract was added Folin–Ciocalteu (150 μL) and Na_2_CO_3_ (120 μL). After that, the solution was added to a microreader plate, and incubated at room temperature for 1 h. After 1 h, the absorbance was measured at 765 nm. The TPC was analyzed using a gallic acid curve and expressed as mg GAE/100 g DW. Reagent blank was also parallelly prepared using ethanol. All the samples were analyzed in triplicate.

### 2.7 Determination of total flavonoid content (TFC)

The total flavonoid contents of sprout extract were analyzed using the respective method of Zhao et al. [30] with few modifications. The sprout extract (250 μL) was mixed with 1.25 μL water and NaNO_2_ aqueous solution (150 μL of 10 wt%). After that, the solution was added to the test tube. Furthermore, water (275 μL) and NaOH solution (0.5 ml 0f 1 M) were added to test tube and mixed properly. The absorbance was measured at 510 nm using a spectrophotometer. Reagent blank was also parallelly prepared using ethanol. The results were expressed as mg CE/100 g DW. All the samples were analyzed in triplicate.

### 2.8 Determination of the radical scavenging activity

The antioxidant activity was determined using DPPH method according to the procedure of Xiao et al. [31] with some modifications. DPPH (7.8 mg) was added to 100 ml ethanol (99.5 %). After that, the DPPH solution was kept in a dark place for 2 hr. The sprout extract (200 μL) was added to 700 μL of Tris-HCl buffer (pH 7.4) and 2000 μl of DPPH solution in a test tube. The solution was mixed and kept in a dark place for 30 min. The absorbance of the extract was measured with spectrophotometer at a wavelength of 517 nm. The blank contained 900 μL Tris-HCl buffer (pH 7.4) and 100 μL ethanol. The antioxidants results were expressed as μmoL TE/100 g DW. All the samples were analyzed in triplicate.

### 2.9 Quantification of phenolic compound through HPLC

For sample preparation, NADES extract was diluted using ultrapure water. The diluted extract was centrifuged at 8000 rpm for 5 min, followed by filtration using PVDF filters of 0.45 µm. For identification of phenolic compounds method followed by Nam et al. [32] was practiced using HPLC (Agilent 1260 infinity) equipped with photodiode array detector (DAD). 5 µL sample volume was injected at a flow rate of 0.5 mL/minute. 5 µL sample volume was injected at a flow rate of 0.5 mL/minute. For phenolic compound deletion, a mobile phase (acetonitrile and 0.1 % formic acid) was used at wavelength (280 to 360 nm). Standard phenolic compounds were used for comparison of retention time to quantify the eluents peaks.

### 2.10 Statistical evaluations

The statistical analysis was measured by Analysis of variance (ANOVA). The data was represented as mean ± SD, and the experimental analysis was performed in triplicates. In this work, simplex lattice mixture design methodology was used to optimize the extraction. The different combinations of different solvents have ranges from 0 % to 100 %. However, the result is always 100 %, when solvents are mixed with various combinations. The simple lattice mixed design was applied using Design Expert 13.0 software. In the system, there were six levels of investigation for each solvent. Table 2 and Fig. 1 demonstrate the 14 experiments that were conducted to investigate the effects of solvent ratios on total phenolic content (TPC), total flavonoid content (TFC) and antioxidant activity (DPPH).

**Table 2:** Results for TPC, TFC and DPPH under mixed design extraction conditions.

**Response**	**Name**	**Units**	**Observations**	**Minimum**	**Maximum**	**Mean**	**Std. Dev.**	**Ratio**
R1	Total phenolic content	mg GAE /100 g	14.00	53.76	128.03	90.89	11.63	1.68
R2	Total flavonoid content	mg CE/100 g	14.00	9.10	38.61	23.85	5.67	1.21
R3	Antioxidant activity	µmol TE/100 g	14.00	1103.5	2117.18	1610.34	4.92	1.15

## 3 Results and discussion

### 3.1 Different experiment conditions and their response

In current research, the simplex lattice mixture design was purposively planned to upgrade the extraction condition to attain best antioxidant recovery, to establish and explain the relationship of responses of interest of the study (TPC, TFC and DPPH assay) as a function of undulations in the proportions of the mixture and to choose the ideal ratios of the mixture based on the criteria demonstrated in Table 3.

**Table 3:** Different experimental conditions of Simplex lattice mixture design and their responses.

		Component 1	Component 2	Component 3	Response 1	Response 2	Response 3
					Total phenolic content	Total flavonoid content	Antioxidant activity
Std	Run	A:Citric acid	B:Glycerol	C:Water	(mg GAE /100 g)	(mg CE/100 g)	(µmol TE/100 g)
11	1	1.00	0.00	0.00	89.19 ± 0.20	9.10 ± 0.10	1109.2 ± 0.50
3	2	0.00	0.00	1.00	90.22 ± 0.30	11.50 ± 0.20	1108.3 ± 0.50
10	3	0.33	0.33	0.33	128.03 ± 0.30	10.70 ± 0.10	2117.1 ± 0.70
13	4	0.00	0.00	1.00	87.16 ± 0.20	10.20 ± 0.10	1108.4 ± 0.50
8	5	0.16	0.66	0.16	74.09 ± 0.20	10.90 ± 0.10	1107.6 ± 0.50
5	6	0.50	0.00	0.50	86.33 ± 0.20	8.55 ± 0.10	1881.2 ± 0.60
1	7	1.00	0.00	0.00	86.37 ± 0.20	9.39 ± 0.10	1110.8 ± 0.50
12	8	0.00	1.00	0.00	86.41 ± 0.20	9.27 ± 0.10	1113.8 ± 0.60
6	9	0.00	0.50	0.50	88.69 ± 0.30	11.40 ± 0.20	1179.4 ± 0.60
2	10	0.00	1.00	0.00	79.31 ± 0.20	29.34 ± 0.20	1108.2 ± 0.50
9	11	0.66	0.16	0.16	84.09 ± 0.20	38.61 ± 0.20	1189.2 ± 0.60
14	12	0.50	0.50	0.00	53.76 ± 0.10	16.90 ± 0.20	1129.2 ± 0.50
4	13	0.50	0.50	0.00	57.04 ± 0.10	14.30 ± 0.20	1127.1 ± 0.50
7	14	0.66	0.16	0.16	73.13 ± 0.10	10.50 ± 0.10	1103.5 ± 0.50

The experimental data was Mathematical modeled by employed mixed design. Even in the absence of an experiment, the model can be used to predict the result at any position within the investigational region of interest because it illustrates the relationship between the solvent proportions and the reaction. The variance proportion that the model explains is reflected in the R^2^ coefficient. Contour plots are the most effective graphical representation of the best mathematical model, enabling the visualization of the measurement of each result under study as a function of solvent mixtures inside the investigational domain.

The TPC, TFC, and DPPH results were optimized simultaneously to optimize the total desirability function. However, findings evaluated that the ternary mixture of citric acid, glycerol, and water in equal quantities (33:33:33 v/v/v) is the best combination for extraction of polyphenols (128.03 ± 0.3 mg GAE /100 g DW) and antioxidant activity (2117.1 ± 0.7 µmol TE/100 g DW). The mixture of tree solvents citric acid, glycerol, and water, in different quantities (66:16:16 v/v/v) was optimal for maximizing flavonoid content (38.61 ± 0.03 mg CE/100 g). Repeatable research was conducted to evaluate the model's prediction capacity under these optimal conditions. The observed values for all replies were within the confidence interval (R^2^ > 0.95) (Tables 2).

### 3.2 Statistical Modeling of the TPC, TFC and DPPH

The TPC, TFC and antioxidants were positively influenced by a combined solvent mixture with a high proportion sprouts ratio. The equations obtained from the three models (TPC, TFC, and DPPH) in terms of the natural components are shown in Table 4. These outcomes indicate that the coefficients determined in the interaction between the chickpea sprout extracts revealed the highest impact.

**Table 4:** Equation of models (TPC, TFC and DPPH) that predicts the relationship between the independent variables and the response variable.

**Response**	**Equation**
**TPC**	TPC = +87.58A + 82.92B + 88.40C-117.22AB-5.05AC + 15.78BC
**TFC**	TFC = +93.04A + 89.83B + 109.53C + 95.72AB-11.61AC + 8.04BC
**DPPH**	DPPH = +109.68A + 111.31B + 108.34C + 74.87AB-105.21AC-110.52BC

The outcomes of analysis of variance (ANOVA) including probability values, regression model terms, R^2^, F-value are provided in Table 5, Table 6, Table 7. R^2^ (the coefficient of determination) was 0.91. The attained value of predicted R^2^ (0.90) was reasonably in agreement with the values of adjusted R^2^ (0.95) of total phenolic content (TPC) assay, the calculated R^2^ value for this assay was 0.97. In case of TFC assay the difference between the predicted R^2^ and adjusted R^2^ value was less than 0.2. The R^2^, adjusted R^2^ and predicted R^2^ of TFC was 0.99, 0.98 and 0.97 respectively. However, R^2^, predicted R^2^ and adjusted R^2^ values of DPPH were 0.98, 0.97 and 0.91 respectively. The current findings illustrated the adequacy of the model to demonstrate the actual relationship among the components. In quadratic model the F-value of TPC (54.41), TFC (167.48), and DPPH (86.36) implied the significance of model. The occurrence of larger F −value had 0.01 % probability that it was because of noise.

**Table 5:** ANOVA table for reduced quadratic model of total phenolic content (TPC).

Source	Sum of Squares	Df	Mean Square	F-value	p-value	
**Model**	1707.47	5	341.49	54.41	<0.0001	significant
(1) Linear Mixture	416.74	2	208.37	33.20	0.0001	
AB	1274.32	1	1274.32	203.02	<0.0001	
AC	1.65	1	1.65	0.2627	0.6221	
BC	16.08	1	16.08	2.56	0.1482	
**Residual**	50.21	8	6.28			
Lack of Fit	10.97	4	2.74	0.2796	0.8776	not significant
Pure Error	39.24	4	9.81			
**Cor Total**	1757.69	13

**Table 6:** ANOVA table for reduced quadratic model of total flavonoid content (TFC).

Source	Sum of Squares	Df	Mean Square	F-value	p-value	
**Model**	994.94	5	198.99	167.48	<0.0001	significant
Linear Mixture	133.60	2	66.80	56.22	<0.0001	
AB	849.73	1	849.73	715.19	<0.0001	
AC	8.70	1	8.70	7.32	0.0268	
BC	4.17	1	4.17	3.51	0.0978	
**Residual**	9.51	8	1.19			
Lack of Fit	3.01	4	0.7514	0.4624	0.7633	not significant
Pure Error	6.50	4	1.62			
**Cor Total**	1004.45	13

**Table 7:** ANOVA table for reduced quadratic model of DPPH antioxidant activity.

Source	Sum of Squares	Df	Mean Square	F-value	p-value	
**Model**	2776.27	5	555.25	86.36	<0.0001	significant
Linear Mixture	579.07	2	289.53	45.03	<0.0001	
AB	519.95	1	519.95	80.87	<0.0001	
AC	714.66	1	714.66	111.15	<0.0001	
BC	788.59	1	788.59	122.65	<0.0001	
**Residual**	51.44	8	6.43			
Lack of Fit	32.27	4	8.07	1.68	0.3131	not significant
Pure Error	19.17	4	4.79			
**Cor Total**	2827.70	13				

Whereas, p-values (p < 0.0001) was an indicative of the accuracy of the model in predicting behavior of the mixtures. The Design Expert software 13.0 was used to conduct ANOVA to analyze the adequacy of the test model. Fitness of models was evaluated by examining statistical parameters like coefficient of determination (R^2^), the coefficient of predicted values and the coefficient of adjusted values, Fisher test (F-value) and *p*-value. High level of significance was demonstrated by all the predicted models and was proven by low *p*-value (*p* < 0.0001).

### 3.3 Effect of solvent mixture on total phenolic content (TPC)

The chickpea sprouts are basically useful and nutritive vegetable [30]. The chickpea sprouts are composed of different types of phytochemicals including proteins, carbohydrates, minerals, fiber, vitamins, health-promoting fatty acids, and phenolic compounds (phenolic acids and flavonoids like primarily *iso*-flavonoids) that play beneficial role in human health [33]. Legume seeds can be processed cheaply and effectively to produce sprouts with nutraceutical potential and improve their nutritional quality [34]. Numerous investigations have been confirmed that bioactive compounds are accumulated during seed germination. However, Gan et al. [35] reported that bioactive substances have antioxidant, anticarcinogenic, antidiabetic, hypolipidemic, and anti-inflammatory properties. Furthermore, edible sprouts may be regarded as foods with possible health benefits [36]. In general, the production and concentration of bioactive substances in seeds can be influenced by germination process. Previous results showed that different are reported to getting seed sprouts for production of more metabolites.

The optimization is performed to find the best extraction condition. Chickpea (*Cicer arietinum* L.) sprouts TPC was examined for various solvent combination. The TPC was changed by the different combination of solvents that are illustrated in Table 3. The TPC range was 53.76 ± 0.1 to 128.03 ± 0.3 mg GAE/100 g DW. The higher value was measured in ternary mixture including: citric acid, glycerol, and water 33:33:33 ratio (v/v/v). The different model was examined to find the regression model that best fits the experimental data. The findings demonstrated that the quadratic model (R^2^ 0.9441) explained a substantial percentage of the variance in TPC response to varying solvent combinations and was significant (*p* < 0.0001). The small misfit (*p* > 0.05) assisted the quality of the model.

The regression coefficients utilized to build a TPC prediction equation are shown in Table 4. The result under study is greatly aided by analyzing the coefficients to consider the impact of every variable. Actually, the weight of each variable is reflected in the coefficient's absolute point, and the mark of the coefficient positive or negative indicates whether the response is affected positively or negatively. While the TPC prediction equation are positive in all the coefficients. However, only the ones corresponding to the mixtures of citric acid, water, and glycerol are significant (*p* < 0.05). This indicates that these solvent combinations have a synergistic response to the separation of polyphenols. Furthermore, the ternary mixture of citric acid, glycerol, and water has the largest correlation. Moreover, its suggested that this solvent combination enhances the extraction of phenolic compounds. The current results support previous research that found moderately polar combinations (citric acid-glycerol-water) are useful for extracting phenolic chemicals from plant protein-rich materials, like chickpeas. The polyphenol–protein complexes may be breakdown due to these mixes [37].

**Fig. 2:**
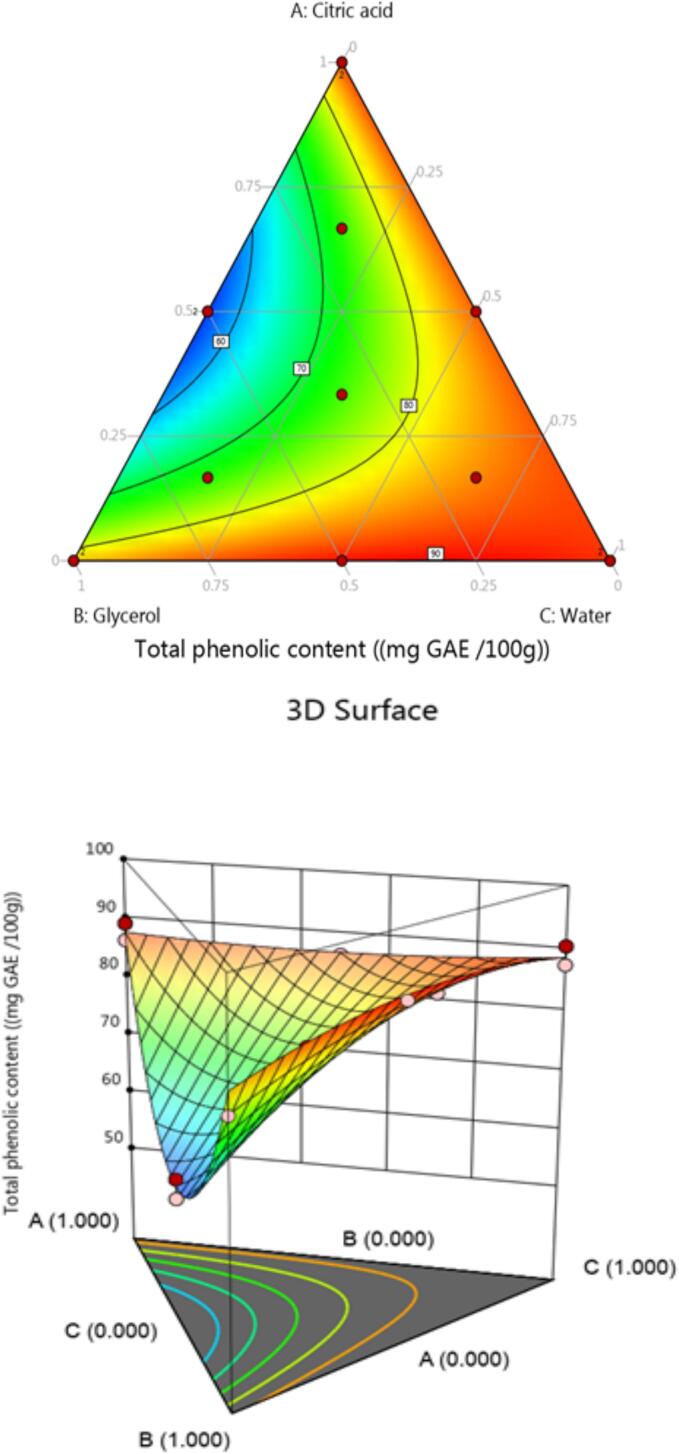
Contour plot and 3D surface graph for TPC.

Different groups of bioactive substances with varying polarity can be found in plant materials. The solubility of polyphenols in a particular solvent is correlated with their individual polarities that clearly affects the extraction efficiency of these compounds. Polar solvents are typically solvents that are choice for phenolic compounds [38]. The solubility is influence by the range of the elemental hydrocarbon series, degree of methoxylation, presence of hydroxyl groups, and molecular size [36]. Contour plots demonstrated that binary and ternary solvents are contained more phenol than pure solvents (Fig. 2). Among all the tested mixtures, pure citric acid extract had the lowest TPC value. It supports the idea that employing pure citric acid encourages its self-association (citric acid–citric acid self-associated molecules). The results lower the likelihood that is associated with the plant matrix compounds for extraction.

Our results showed the higher TPC values ​​of chickpea (*Cicer arietinum* L.) sprouts using NADES ternary mixture extract (128.03 ± 0.3 mg GAE/100 g DW) whereas previous study showed the lower value using the solution containing different combinations of hydrogen peroxide (H_2_O_2_) [33]. Another study reported by Hayta & Isçimen, [39] measured the lower value of total phenolic compounds (14.77 mg GAE/mL extract) using water extract. The results showed that current study have significantly more TPC than water extract.

Furthermore, no data has been published on extracting the total phenolic compounds from chickpea sprouts using NADES as a solvent. Our results are compared with previous studies that performed on legumes sprouts using traditional organic solvents and water [40], mung bean sprouts methanol extracts [44], soybean sprout ethanol [42], *Chenopodium quinoa* wild methanol extract (Quinoa) [43], and buckwheat (*Fagopyrum*) sprouts methanol extract [44].

Previous work demonstrated that the synergistic effect of combinations of water and naturally occurring deep eutectic solvent chemicals (citric acid and glycerol) for the extraction of phenolic compounds from various plant matrices [45]. The natural solvent (water) has the capacity to expand cells and break down their walls, which increases the penetration of natural deep eutectic solvent chemicals inside cells [46]. Additionally, phenolic chemicals can be dissolved in water and frequently conjugated with other molecules including proteins and polysaccharides.

Additionally, the density and viscosity of a solvent can have a significant impact on its extraction capacity [47]. Because of their high diffusivity and the importance of solvent and solute molecular mobility in enhancing extraction efficiency, low viscosity and density solvents typically have high extraction potential [48]. The extraction of phenolic compounds is positively impacted by the combination of water and natural deep eutectic solvent components because this combination lowers the solvent systems density and viscosity values, enhancing its diffusivity and streamlining the extraction process.

### 3.4 Effect of solvent mixture on total flavonoid content (TFC)

The TFC value of chickpea (*Cicer arietinum* L.) sprouts extract was ranged from 38.61 ± 0.2 to 9.10 ± 0.1 mgCE/100 g DW at different combination NADES solvent. The mixture of different solvents including water, citric acid, and glycerol was shown to have a high affinity for the flavonoids by the quadratic model. The higher flavonoid concentration of chickpea (*Cicer arietinum* L.) sprouts was 38.61 ± 0.2 mg CE/100 g DW. The results showed that TFC concentration was increased with increase of citric acid in the combination (Table 3). The optimal amount ratio of solvents was citric acid, glycerol, and water: 66:16:16 ratio (v/v/v). In contrast, the number of flavonoids increased with the increase the amount of citric acid.

The addition of the appropriate amount of water is causes the flavonoid molecules in the NADES to dissolve more readily because it decreases the molecular interaction. In this work, the flavonoid extraction values were achieved using the ternary mixing of NADES solvents which is related to the values obtained in previous studies using traditional organic solvents. In our study, the higher TFC value of chickpea sprouts using NADES ternary mixture extract was 38.61 ± 0.2 mg CE/100 g DW, which was greater than the values found in previous study using DESs (choline chloride/ propylene glycol) extract of chickpea sprouts [49]. Another study was conducted by León-López et al. [33] in the different combinations of hydrogen peroxide (H_2_O_2_) extract of chickpea Sprouts. The results reported the low amounts of total flavonoids as compared to current results.

On the other hand, the previous study was conducted on lentil sprouts using aqueous extract. The TFC value was several times lower than our results [40]. Kim et al. [44] performed a study on buckwheat (*Fagopyrum*) sprouts using water as a solvent. The finding showed lower value of TFC as compared to current finding. In the literature, we did not find data on the extraction of total flavonoids from chickpea (*Cicer arietinum* L.) sprouts using the NADES solvent. Our results compared with other study that found that DES based on ethylene glycol-glycolic acid was more effective in extracting flavonoid from mung bean [50].

**Fig. 3:**
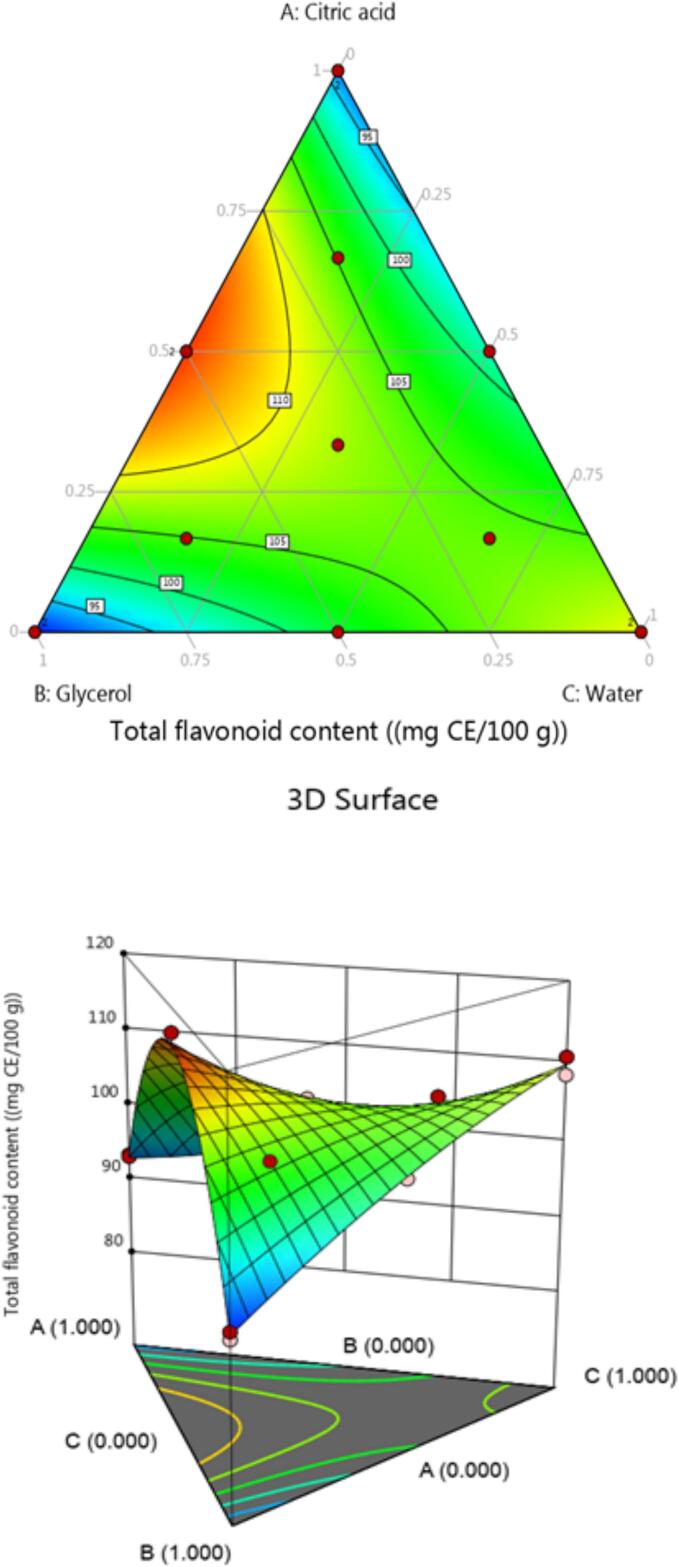
Contour plot and 3D surface graph for TFC.

For the variance of analysis, there are four optimization models were analyzed including cubic, linear, special cubic and quadratic (Fig. 3). The same models were applied to phenolic compounds. The quadratic model proved to be the most efficient concerning to Table 6. This model was significant (*p* < 0.0001) and had the highest determination coefficient and adjusted determination coefficient.

### 3.5 Effect of solvent mixture on antioxidant activity by DPPH

Antioxidants are substances that can prevent or slow down the oxidation process that cause by free radicals. However, the organism may experience oxidative stress which can impair physiological processes. The rate at which free radicals are produced that are covered by antioxidants. Natural molecules that may fall in the many chemical classes including phenolic compounds are known as antioxidants. However, these are often too common class and are well-known for being potent antioxidants because of their capacity to transfer electrons, hydrogens, or chelate metals. The antioxidant activity of chickpea (*Cicer arietinum* L.) sprouts was evaluated using the DPPH that is completely comprehend the solvent effect on the antioxidant activity. The antioxidants were ranged from 1103.5 ± 0.5 to 2117.1 ± 1.8 µmol TE/100 g DW. The mixture of citric acid, glycerol, and water: 33:33:33 ratio (v/v/v), had the maximum antioxidant activity whereas, the mixture of citric acid, glycerol, and water: 66:16:16 ratio (v/v/v) showed the lowest antioxidants activity (Table 3).

After analyzing the developed regression models, it was determined that the full cubic and special cubic models did not significantly increase the fit for the surface. However, quadratic model was selected for the antioxidant test. Moreover, all quadratic models were analyzed significantly concerning the R^2^ range from 0.9704. However, the model did not exhibit a substantial lack of fit at the 95 % confidence level. The regression coefficients for the measurement of the antioxidant equation are shown in Table 4. For the various solvent mixes, there was no discernible antagonistic impact. However, certain combinations were more synergistic than others. It can be attributed to the combined impact of the citric acid-glycerol-water which demonstrated the highest regression coefficient across all the tests for antioxidant activity. The extract of the ternary combination contained more antioxidant activity as compared to the pure solvent.

**Fig. 4:**
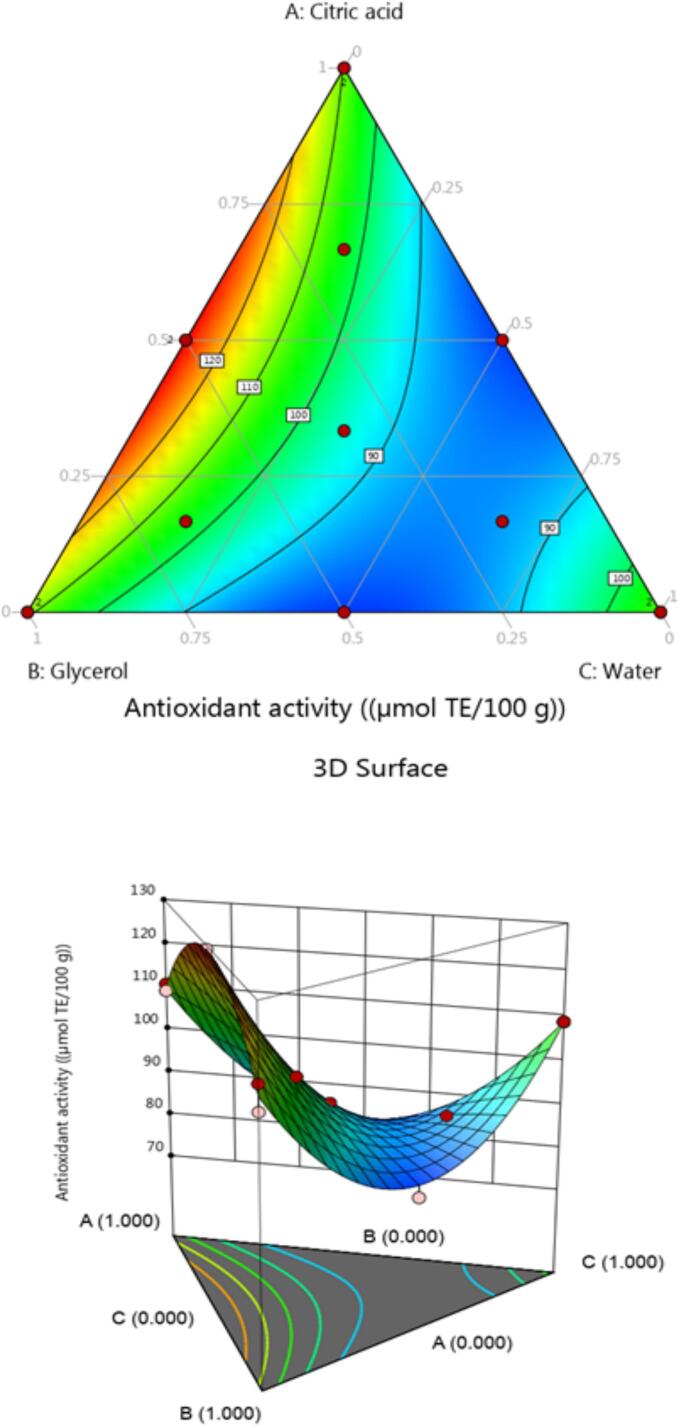
Contour plot and 3D surface graph for antioxidant activity by DPPH.

The contour plots of the antioxidant activity test (Fig. 4) show that these all followed the same pattern as phenolic chemicals (Fig. 2). There is a substantial association (*p* < 0.05) between phenolic component concentration and antioxidant activity. The characterization of phenolic compounds based on structure has a direct impact on the antioxidant capacity. However, this capacity is due to the position and number of the hydroxyl group, distance between the carbonyl group and the aromatic ring, the number of aromatic rings, the extent of conjugation, and the degree of methoxylation [51].

The Chickpea (*Cicer arietinum* L.) sprouts have higher antioxidant activity than previous work performed with traditional organic solvents [33], [41], [43], [44], [52]. These results indicate that the NADES technique can be used to extract anthocyanins and other sustainable phenolic chemicals instead of organic solvents. Antioxidants are also extracted for the polyphenols that contribute to the activity. Furthermore, the phenolic compounds extracted by ternary mixture may have more antioxidant activity. These ternary graphs are served as a visual representation of the predictive model. It is demonstrated that the citric acid-glycerol water mixture (33:33:33) is responsible for the greatest TPC, and antioxidant activity.

Furthermore, the NADES-based solvent extracts could be added directly into food products without any requirements of post-extraction purification. Moreover, this extract may be beneficial in the formation of functional food products. Overall, it is showed significant antioxidant activity. Chickpea (*Cicer arietinum* L.) sprouts are a byproduct of the food industry that can be grown sustainably and employed in pharmaceutical and cosmetic products.

### 3.6 Characterization of the phenolic profile of chickpea (Cicer arietinum L.) sprout

**Fig. 5:**
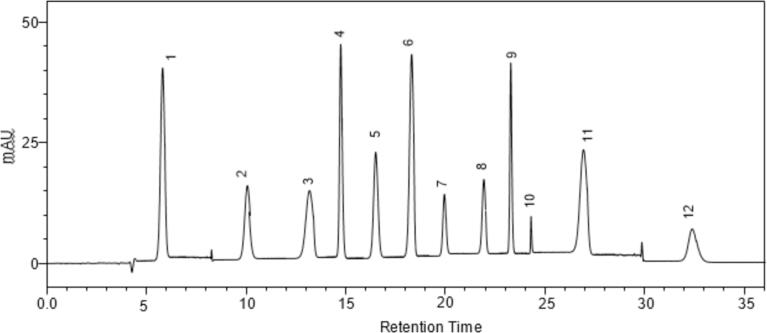
Phenolic compounds profile from HPLC-DAD analysis at 280–360 nm, NADES extract. Peaks’ identification: 1-chlorogenic acid; 2-catechin; 6-epicatechin; 7-syringic acid.

Using HPLC equipped with diode array detector (HPLC-DAD) High-performance liquid chromatography with diode-array detection (HPLC-DAD) at 280––360 nm wavelength similar phenolic acid profile of chickpea (*Cicer arietinum* L.) sprout NADES extract was attained (Fig. 5). The finding of the test exhibited that chickpea sprout contains a diverse range of phenolic compounds. The eluted compounds were identified and quantified through comprehensive and efficient chromatograph analysis of phenolic compounds. The NADES ternary mixture exhibited a significantly varying range of intriguing phenolic components when compared to ethanol, the reference organic solvent fraction.

The identified phenolic compounds and their related metabolite fractions were nearly correlated to outcomes of ternary mixture of the NADES and ethanolic extracts. Whereas, the recovered quantity of polyphenols was significantly higher when organic solvent was used. Similar result was attained by Nam et al. [32] while studying phenolic compounds of buckwheat sprouts; in methanol fraction of buckwheat sprout higher concentration of rutin (5.5 mg/g DW) was attained as primary component of the fraction. Magalhães et al. [52] observed 45.9 mg/kg syringic acid using 1:1 methanol: water (v/v) binary solvent from chickpea sprout extract.

The findings of current study demonstrated the highest concentration polyphenol catechin i.e., 8.25 mg/g DW which was the most abundant and prevalent of all phenolic compounds in NADES extracts. Other attained phenolic fractions were, coumaric acid (0.02 mg/g DW), epicatechin (3.15 mg/g DW), rutin (0.90 mg/g DW), ferulic acid (0.03 mg/g DW), gallic acid (0.76 mg/g DW), kaempferol 3-glucoside (0.06 mg/g DW) ferulic acid (0.03 mg/g DW), chlorogenic acid (7.25 mg/g DW) and syringic acid (1.20 mg/g DW).

The outcomes of the current study proved the effectiveness of the NADES system as a substitute of organic solvent, used for isolation of phenolic components from *Cicer arietinum* sprout. In food industry application of GRAS NADES solvents like combination of water, citric acid and glycerol will have significant impact. These NADES are an environmentally friendly, viable and sustainable alternatives to organic solvents, conventionally used for phenolic compound extraction. These finding not only promote sustainability but also have significantly advanced potential to manage the specific purpose of phenolic compound extraction, like its utilization as an alternative to conventional antioxidants that are used to improve the nutritional quality of different products of food industry. Furthermore, our research also demonstrated that for experimental setting organization and optimization simplex lattice mixture design proved to be a powerful and efficient methodology to achieve optimal outcomes with minimal experimentation.

## 4 Conclusions

It is concluded that chickpea sprouts are composed of bioactive compounds including antioxidants, phenolic compounds and total flavonoids. However, the high number of bioactive compounds is measured in the sprout extract using natural solvent combinations. Furthermore, green techniques (ultrasound and NADES) are found to be best for the extraction of bioactive compounds from sprouts. The higher value of antioxidants and phenolic compounds were observed in the solvent mixture of citric acid, glycerol and water; 33:33:33 (v/v/v). Moreover, the higher value of total flavonoids was found in the solvent mixture citric acid, glycerol and water; 66:16:16 (v/v/v). The findings showed that a mixture of natural solvents is the best alternative to organic solvents for the sustainable extraction of polyphenols. However, these findings may be most advanced in managing the extraction of polyphenols for specific purposes such as the replacement of conventional extraction methods for the food sector to improve stability. Furthermore, our findings show that the current design is a valuable and effective procedure for developing and refining experiments to attain good findings using limited conditions. In the future, the NADES extraction method can be bright because it can improve the sustainability of industries by utilizing processing byproducts and replacing organic solvents with safe GRAS ingredients. The potential for NADES extraction in the food industry is encouraging since it might enhance the sustainability of the sector by utilizing processing by-products and substituting organic solvents with renewable GRAS components. In the future, the NADES extraction method can be bright because it can improve the sustainability of industries by utilizing processing byproducts and replacing organic solvents with safe GRAS ingredients.

## CRediT authorship contribution statement

**Waseem Khalid:** Writing – review & editing, Writing – original draft, Software, Investigation, Formal analysis, Data curation. **Hyrije Koraqi:** Writing – review & editing, Visualization, Validation, Resources, Formal analysis, Conceptualization. **Imed E Benmebarek:** Writing – review & editing, Visualization, Validation, Supervision, Project administration, Methodology, Formal analysis. **Andrés Moreno:** Writing – review & editing, Writing – original draft, Visualization, Validation, Supervision, Project administration, Methodology, Investigation, Formal analysis, Data curation, Conceptualization. **Tawfiq Alsulami:** Writing – review & editing, Supervision, Resources, Methodology, Funding acquisition, Formal analysis, Data curation, Conceptualization. **Robert Mugabi:** Writing – review & editing, Software, Methodology, Funding acquisition, Formal analysis, Data curation. **Gulzar Ahmad Nayik:** Writing – review & editing, Visualization, Software, Resources, Formal analysis, Data curation.

## Declaration of competing interest

The authors declare that they have no known competing financial interests or personal relationships that could have appeared to influence the work reported in this paper.
